# A macroevolutionary analysis of European Late Upper Palaeolithic stone tool shape using a Bayesian phylodynamic framework

**DOI:** 10.1098/rsos.240321

**Published:** 2024-08-14

**Authors:** David N. Matzig, Ben Marwick, Felix Riede, Rachel C. M. Warnock

**Affiliations:** ^1^ Department of Archaeology and Heritage Studies, Aarhus University, Højbjerg, Denmark; ^2^ Department of Anthropology, University of Washington, Seattle, WA, USA; ^3^ GeoZentrum Nordbayern, Friedrich-Alexander-University Erlangen, Erlangen, Germany

**Keywords:** cultural macroevolution, Bayesian phylogenetics, geometric morphometrics, archaeology, Late Upper Palaeolithic, stone tools

## Abstract

Phylogenetic models are commonly used in palaeobiology to study the patterns and processes of organismal evolution. In the human sciences, phylogenetic methods have been deployed for reconstructing ancestor–descendant relationships using linguistic and material culture data. Within evolutionary archaeology specifically, phylogenetic analyses based on maximum parsimony and discrete traits dominate, which sets limitations for the downstream role cultural phylogenies, once derived, can play in more elaborate analytical pipelines. Recent methodological advances in Bayesian phylogenetics, however, now allow us to infer evolutionary dynamics using continuous characters. Capitalizing on these developments, we here present an exploratory analysis of cultural macroevolution of projectile point shape evolution in the European Final Palaeolithic and earliest Mesolithic (approx. 15 000–11 000 BP) using a Bayesian phylodynamic approach and the fossilized birth–death process model. This model-based approach leaps far beyond the application of parsimony, in that it not only produces a tree, but also divergence times, and diversification rates while incorporating uncertainties. This allows us to compare rates to the pronounced climatic changes that occurred during our time frame. While common in cultural evolutionary analyses of language, the extension of Bayesian phylodynamic models to archaeology arguably represents a major methodological breakthrough.

## 1. Introduction

Phylogenies are a vital tool in palaeobiology for understanding the patterns and processes of organismal evolution, diversification, extinction and adaptation [1,2]. The introduction of formal cladistics in the 1960s [3], ever-increasing computing power [4,5] and more recent introductions of powerful Bayesian statistical frameworks [6–9] have had a profound impact on the quantitative rigour and reproducibility of palaeobiological analyses. In the human sciences, the use of phylogenetic methods to reconstruct and analyse historical ancestor–descendant relationships based on cultural traits on the whole lags somewhat behind in terms of the breadth of application and its analytical sophistication. Notably, linguists have long been aware of the similarities between language trees and organismal phylogenies [10,11]. Consequently, and thanks to the systematic compilation of discrete lexical data, phylogenetic analyses [12–14]—including those using Bayesian methods—are now commonplace in historical linguistics, and have made singular appearances in other disciplines related to cultural evolution, such as musicology [15].

Archaeology is another human science in which phylogenetic analysis has gained some traction in recent decades. Spurred by the development of, initially, the notion of the extended phenotype and later the emergence of cultural evolutionary theory [16,17], archaeologists have adopted phylogenetic methods to model and understand material culture evolution [18–26]—Bayesian phylogenetic methods, however, remain the exception [27–31]. Humanly made objects—artefacts—can be described in many ways and archaeologists have devised countless, often not readily compatible qualitative and quantitative ways of doing so. Chief among these is typology, a pre-computational and essentialist classification practice that partitions variation into presumed average forms [32,33].

Inspired by the debate about unit definition in biology (e.g. [34,35]), modern artefact phylogenetics has attempted to break with this classificatory approach, arguing that partitioning individual artefacts into a collection of traits that together describe them circumvents the unduly reifying tendencies of typological classification (cf. [36]). In doing so, however, continuous characters are commonly discretized into arbitrary bins that also do not fully describe the observed variation (e.g. [33]). Again inspired by palaeobiology, archaeologists have also employed landmark-based geometric morphometrics (GMM) to describe artefact shapes (e.g. [37,38]). While this mitigates the drawbacks of using qualitative or discretized traits, landmark placement on artefacts nonetheless remains difficult [39]. Most recently, approaches using whole-outline GMM—which is entirely independent of landmark placement and richly describes artefact shape—have been benchmarked against previous studies [40]. While the conceptual basis for using such continuous characters (e.g. principal components) in phylogenetic analyses has been firmly established [41–45], pipelines for actually doing so within a Bayesian framework have hitherto been lacking [46–48], stymieing both organismal and artefactual phylogenetics. Fresh developments in Bayesian phylogenetics—specifically the development of the computational environments of BEAST2 [8] and RevBayes [9]—now offer flexible analytical pipelines that facilitate the construction of phylogenies from continuous characters (e.g. [49]). Furthermore, we now have statistically coherent models for the analysis of sampling data through time.

Models that combine diversification and sampling processes have also undergone massive development over the past decade. Within palaeobiology, a significant step forward was the introduction of the fossilized birth–death (FBD) process [50,51]. This model explicitly includes the fossil sampling process, which accounts for the possibility that extinct samples can appear in the tree as tips or samples along internal branches (sampled ancestors). The FBD process has already been applied to study language evolution (e.g. [52]), and here, we apply this model for the first time within an archaeological context. The use of *phylodynamic models* further expands the information we can obtain from phylogenetic trees. While phylogenetics seeks to estimate the evolutionary relationships among samples, phylodynamics seeks to estimate variation in the dynamics that generated our trees [53]. For example, skyline birth–death process models allow for variation between discrete time intervals in the diversification and sampling rates [54]. Applied within a Bayesian framework, we can account for uncertainty in the tree topology and divergence times, meaning we can characterize the diversification dynamics without perfect knowledge of the underlying phylogeny. Phylodynamics is often applied in the context of epidemiology where, for example, low mutation rates might prevent us from inferring a fully resolved transmission tree, but we can still estimate variation in transmission rates [55]. In the context of macroevolution, we can apply the FBD skyline model to obtain estimates of speciation, extinction and fossil sampling rates through time [56].

**Figure 1.**
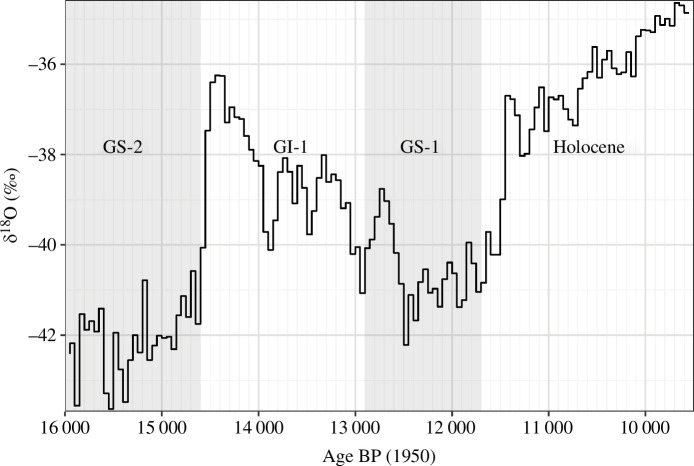
Fifty-year means of NGRIP $\delta^{18}O$ levels in per mille for the time frame in question together with the Dansgaard–Oeschger Events highlighted in grey. A 1‰ change corresponds to a *ca* 3°C change in temperature [57]. GS−2: Greenland Stadial 2, *ca* 22.9–14.7 ka BP; GI−1: Greenland Interstadial 1, *ca* 14.7–12.9 ka BP; GS−1: Greenland Stadial 1, *ca* 12.9–11.7 ka BP; Holocene, from *ca* 11.7 ka BP.

Our study reports on the very first such application of phylodynamics to archaeological data, specifically to knapped stone projectile points from the European Late Upper Palaeolithic (LUP). The European LUP (*ca* 15–11.5 ka BP) falls into a time of extremely volatile climate and changing environments. The period starts at the end of the Last Glacial Maximum (GS−2), which is followed by a phase of abrupt warming (GI−1), in which icesheets regressed significantly, new landscapes opened up, and flora and fauna underwent considerable changes. Following one-and-a-half millennia of intermittent warming, this interglacial was cut short by the onset of Greenland Stadial 1 (GS−1) at approximately 12.9 ka BP, with a deterioration in climate and the return to very cold, dry and windy conditions, particularly in northern Europe. With the termination of this stadial at approximately 11.7 ka BP, the Pleistocene ended, and the Holocene began (figure 1; [57]).

Archaeologists have long studied the impact of climatic changes on human communities during this time (e.g. [58–60]). In comparison with preceding periods of the European Upper Palaeolithic—the iconic Magdalenian—it is argued that the LUP underwent both simplifications of and losses in technology [61] as well as, eventually, a diversification of regional forms. Such assessments are, however, derived chiefly from studies conducted at the scale of sites or regions. Yet, it is arguably at the macro-scale at which the archaeological record can truly shine (e.g. [62]). In fact, previous studies comparing LUP cultures and artefact forms quantitatively across European regions have already shown that many traditional archaeological cultures are beset by issues of epistemic or empirical inadequacy (e.g. [63–66]).

Despite the evident benefits of macro-archaeological studies—especially when combined with cultural evolutionary theory—these remain rare [67]. The often regionally bespoke and mutually incompatible classifications of archaeological material constitute a major barrier to trans-regional, macro-scale studies. The complex socio-political landscape of past and present Europe further complicates the situation, with the result being a patchwork of archaeological cultures whose analytical utility varies [64,68]. A recent initiative has tested the robustness of these existing archaeological cultures empirically [66], and in doing so, collated an extensive dataset covering many key sites of the European LUP [69]. Our analysis draws on this dataset, but we restrict our analysis to a highly resolved subset of chronologically constrained artefacts, namely projectile points, from such key sites. Based on the tanged/stemmed design of these projectile points, it is reasonable to assume that they were axially hafted and that they served as the working end of hunting weapons that were ostensibly instrumental in the adaptations of these Terminal Pleistocene communities, and that the specifics of their design also reflect aspects of cultural transmission. In focusing on artefact form, we do not deny the utility of discrete (often qualitative) traits—motivated by and derived from detailed technological analyses (cf. [70,71])—in building artefact phylogenies. Large and representative datasets of such traits tested for intra- and inter-observer bias do not exist at present, however. Moreover, artefact shape remains one of the critical phenomenological traits used to distinguish many, if not most, projectile types, underlining the powerful yet mostly untested role that this trait alone plays in traditional cultural taxonomy.

Building on previous work [49], we here take initial exploratory steps towards the Bayesian phylodynamic inference of stone tool phylogenies. A vital contribution here lies first in the benchmarking of these computationally demanding methods, the different models, the data and their interaction. Second, inferring a Bayesian phylogeny from artefact shapes directly without any *a priori* cultural associations opens up the possibility for the macro-scale study of cultural evolution, including a direct quantitative assessment of the rates of artefact lineage diversification and extinction, and morphological disparity [72]—especially so across the climatically volatile periods of the Terminal Pleistocene, across the many transitional cultural phases, and across the many topographically and biogeographically variable regions of Europe. Our results offer new data-driven insights into the cultural evolutionary dynamics at the end of the Pleistocene in Europe. Notably, the resulting artefact phylogeny maps onto patterns of human dispersal where isolation by distance and climate pressure result in diversification, whereas periods of climatic amelioration and population growth appear to be characterized by a loss of diversity in artefact forms. Our results also align, albeit not perfectly, with recent palaeogenomic results [73] that suggest relations between southern and central Europe.

## 2. Material and methods

### 2.1. Artefacts and outline data

**Figure 2.**
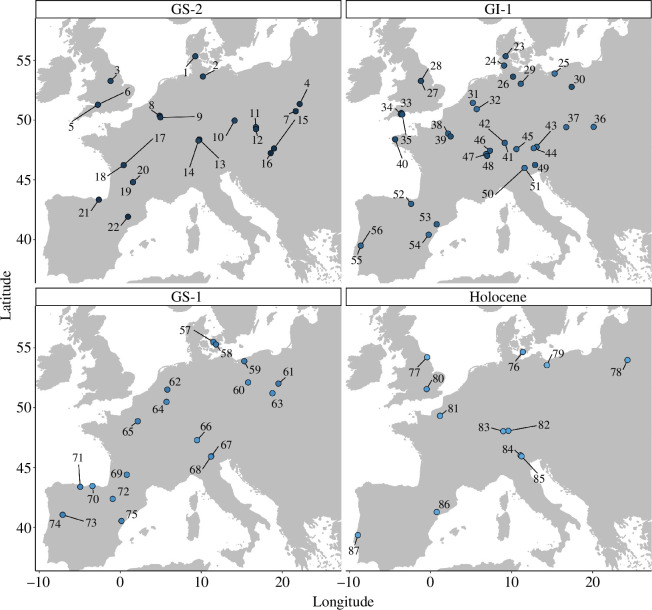
The study region with available key site/layer combination per time bin, showing today’s coastlines. The map is chronologically separated according to the Dansgaard–Oeschger Events [57] into Greenland Stadial 2 (GS−2), Greenland Interstadial 1 (GI−1), Greenland Stadial 1 (GS−1) and Holocene. 1: Slotseng C, 2: Poggenwisch, 3: Pin Hole Cave, 4: Klementowice 20, 5: Sun Hole, 6: Gough’s Cave, 7: Wilczyce 10, 8: Bois Laiterie, 9: Trou de Chaleux, 10: Hostim, 11: Kulna 6, 12: Pekarna, 13: Helga Abri, 14: Felsställe, 15: Zöld, 16: Nadap, 17: Bois Ragot, 18: Bois Ragot 5, 19: Murat, 20: Murat, 21: Santimamine Slnc, 22: Parco II, 23: Slotseng B, 24: Ahrenshöft LA 58d, 25: Rotnowo 18ll, 26: Meiendorf, 27: Mother Grundy’s Parlour, 28: Robin Hood’s Cave, 29: Weitsche, 30: Mirkowice 33, 31: Westelbeers ZW, 32: Rekem, 33: Pixie’s Hole, 34: Three Holes Cave, 35: Kent’s Cavern, 36: Nowa Biala 1, 37: Kulna 4, 38: Le Closeau 4, 39: Étiolles Q31, 40: Rocher de l’Impératrice, 41: Zigeunerfels FG, 42: Zigeunerfels DE, 43: Elsbethen, 44: Unken, 45: Seewände, 46: Abri Neumühle, 47: Grotte de Bichon, 48: Hauterive-Champréveyres, 49: Grotta Clusantin, 50: Riparo Dalmeri 15, 51: Riparo Dalmeri (upper layers), 52: Anton Koba VIII, 53: Hort de la Boquera, 54: Roureda, 55: Lapa dos Coelhos 4, 56: Lapa dos Coelhos 3, 57: Bromme, 58: Trollesgave, 59: Rotnowo 18, 60: Wojnowo Acut III 75, 61: Witow 5C (assemblage 1), 62: Milheeze Hutseberg 2, 63: Kochlew 1, 64: Remouchamps, 65: Le Closeau 14, 66: Altwasser, 67: La Cogola SU 19, 68: Palu Echen, 69: Port de Penne, 70: Perro 2, 71: Los Azules Unit 3, 72: Peña 14, 73: Fariseu 4, 74: Fariseu SU 4, 75: Cingle de l’Aigua, 76: Syltholm VII, 77: Star Carr, 78: Kabeliai 2, 79: Bolkow 1, 80: Three Ways Wharf C West, 81: Alizay, 82: Bad Buchau Kappel, 83: Jägerhaushöhle Schicht 13, 84: Romagnano III (Layer A-E), 85: La Cogola SU 18, 86: Filador 4, 87: Bocas 0.

We draw upon a published dataset [69] containing European Late Palaeolithic/earliest Mesolithic lithic technology, toolkits and artefact shapes together with their associated metadata. This dataset had been designed for the study of stone tool technology and artefact shape evolution across Europe for the time frame of approximately 15–11 ka BP. It contains, among other data, the two-dimensional outline shapes of 3512 expert-sourced projectile points stemming from key archaeological sites (figure 2).

Lithic projectile points have long played a major role in the periodization of the Late Palaeolithic/earliest Mesolithic of Europe, and in inferring the ‘ethnogeographic variability’ [74] of presumed contemporaneous communities. Likewise, they are commonly linked to specific hunting strategies, and changes in projectile point forms are interpreted to index changing adaptations. By the same token and with reference to ethnographic observations that link different projectile point shapes to community identities (e.g. [75–77]), lithic point shapes are used by many archaeologists as paradigmatic cultural markers in much the same way that index fossils are used in biostratigraphy. These insights are supplemented with archaeological reconstructions of social learning scenarios that arguably document the transmission of knowledge and know-how from elders to youngsters in this period [78–81]. Through meticulous refitting and technological reverse engineering, these studies suggest that particular stone-working recipes—operational schemata—were passed from generation to generation [82]. In this way, artefact shapes may reflect parts of such recipes and can hence be used to infer transmission histories [33,83,84]. In contrast to many lithic artefacts, whose evolution has hitherto been addressed using phylogenetics, the European LUP stone points appear to have been expedient components in the total weapon system; they were replaced rather than resharpened upon breakage.

**Figure 3.**
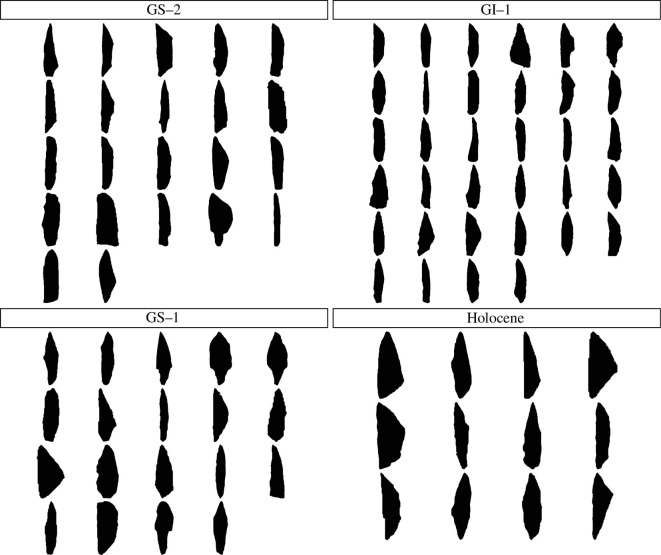
Panel visualizing the 87 selected artefacts, chronologically separated according to the Dansgaard–Oeschger Events (see figure 1). The artefacts have been subsampled from the database published in [69], reoriented, centred and scaled. Each of the artefacts represents one unique key site/layer combination for which radiocarbon dates were available. In this visualization, the scales vary between the sub-plots.

After a thorough quality assessment of those shapes, we retained a subset of 985 complete armatures—overwhelmingly unifacial projectile tips manufactured on blades—from sites for which radiocarbon dates are available and whose dating quality was deemed ‘reliable’ (i.e. dates derived from stratified, *in situ* layers, in the [69] dataset). For each unique key site/layer combination with radiocarbon dates available, we randomly drew one single specimen, resulting in 87 artefacts representative of the whole research area (figure 3). This way of subsampling reflects the assumptions of the FBD process, assuming a Poisson sampling process along each lineage or branch. This means that a given lineage can only be sampled once at any given time point. All artefacts from the same unique key site/layer combination can be considered equivalent to abundance data in palaeontology. This means we have multiple individuals per lineage at a given locality/layer, corresponding to a single point or short interval in time.

**Figure 4.**
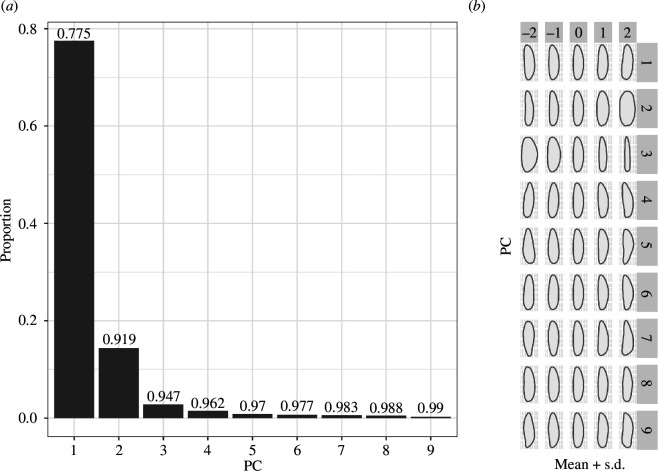
(*a*) The cumulative percentage of variance explained by each individual PC axis. Here, the first nine PC axes capture 99% of the total shape variation. (*b*) The shape variation along the PC axes.

Using the R [85] package Momocs [86], we extracted the two-dimensional whole outlines from all 87 artefacts and combined them with their metadata. As necessary, artefacts were manually reoriented to a standardized direction, and the outlines were centred and scaled. Elliptic Fourier transformation was applied to describe the artefacts’ shape contours in harmonics. The first point of the outlines was set to be homologous. We used 28 harmonics to describe 99.9% of the harmonic power. These data were then transferred via a principal components analysis (PCA) for dimensionality reduction to arrive at new, de-correlated variables. This resulted in 87 principal component axes to describe 100% of the outlines’ variation (figure 4*a*). Figure 4*b* shows the specific shape aspect captured by the first nine PC axes.

### 2.2. Radiocarbon dates

**Figure 5.**
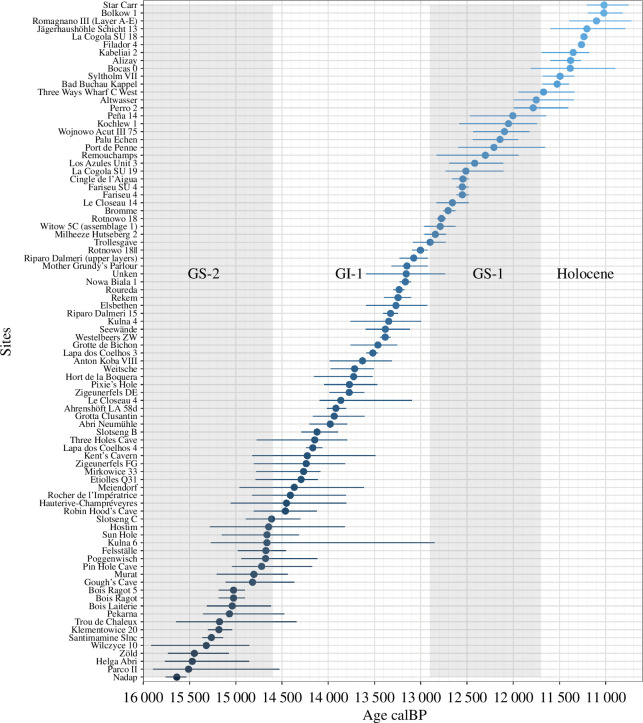
All selected key site/layer combinations with their associated median ages (dots) and age uncertainties (horizontal lines). Each date is derived from the calibrated SPD of all available radiocarbon dates for each site/layer combination. For models with age uncertainty, the one-sigma ranges of the SPDs were taken as the minimum and maximum ages. Otherwise, the median ages of the SPDs were used.

Radiocarbon dates for the sites were collected from the Radiocarbon Palaeolithic Europe Database [87], and other site-relevant literature cited in the original dataset [69]. All radiocarbon dates were inspected for reliability both in the context of the original data compilation [69] and again for the present paper. The dates were then calibrated in R using the rcarbon package [88] and the IntCal20 calibration curve [89]. For each unique key site/layer combination, we calculated the summed probability distributions (SPDs) using the same package. For models without age uncertainty, the median age of the calibrated SPD was used as the single age to which each key site/layer should be dated. For models with age uncertainty, described below, the minimum and maximum of the one-sigma region of the calibrated SPD was used for the range of uncertainty for each key site/layer’s date (figure 5).

### 2.3. Phylodynamic inference

One of the most important advantages of a Bayesian phylodynamic framework is its flexible modular model-based nature. For inferring dated trees and evolutionary rates, we can use a tripartite model [6,90,91], which combines a character, clock and tree model within a single Bayesian analysis. The character model provides a model of phylogenetic character or trait evolution and is needed to calculate evolutionary distances or disparity. The clock model describes how the substitution rate varies across the tree. The tree (phylodynamic) model describes the process of branching (birth), extinction (death) and lineage sampling that generated the tree. It is the tree model that incorporates age information, which, combined with the clock model, allows us to transform evolutionary distances into time. We can also estimate the phylodynamic parameters associated with the tree model, including variation in rates through time.

#### 2.3.1. Tree model

For our tree model, we chose the FBD process [50,51]. We estimated a time-calibrated tree using the Bayesian phylogenetic software BEAST2.6 [8], using the FBD tree model, which combines the diversification (birth, death) and sampling processes. The FBD model allows for the explicit incorporation of fossil samples, i.e. specimens sampled before the present (*t* = 0). Samples with *t* > 0 can either appear on terminal branches (tips) or along branches as sampled ancestors, and their placement can be estimated during inference using the sampled ancestors (SA) package [56]. The model can be used for phylogenetic tree inference of extinct and extant samples or extinct samples only. The FBD process allows us to use the age information of all artefacts and jointly estimate trees and diversification rates. We used the ‘canonical’ parametrization, with priors on the birth, death and sampling rate parameters. For each of these parameters we use an exponential prior, *Exp*(10), with a mean of 0.1. The birth rate (λ) is the rate at which new lineages are added to the tree. The death rate (μ) is the rate at which lineages are terminated. The fossil sampling rate (ψ) is the rate of sampling-through-time along lineages, i.e. for samples, *t* > 0. An exponential distribution places most of the prior probability on values close to zero but the diffuse tail of the distribution does not preclude much larger values. This choice was based on the order of magnitude of rates that are typically estimated in the context of macroevolution (e.g. [92–94]). We set the extant species sampling probability (ρ) to zero, as all our samples existed before the present (*t* > 0). We used a uniform prior on the origin time of the process, *U*(0, ∞).

#### 2.3.2. Character evolution model

Continuous traits have been widely used for phylogenetic comparative analyses (i.e. studying trait evolution along an existing time-calibrated tree) and have more recently also been used for tree inference and divergence dating [47]. For BEAST2, the contraband package v. 0.0.1 was recently developed [95], which we deployed to use a multivariate Brownian motion model to infer the evolution of the continuous shape traits under the assumption of a random walk. This model has three parameters that we estimated: the root values for each trait, the variance (or rate) parameter of the Brownian motion process for each trait ($\sigma^{2}$), and the among-character covariance. For the prior on the root values, we used a normal distribution *N*(0, 2), for the variance parameter $\sigma^{2}$, we used a lognormal distribution *LN*(1, 0.3), and for the covariance parameter we used a uniform distribution, *U*(−1, 1), following the examples in [95].

#### 2.3.3. Clock model

For the clock model, we experimented with two separate relaxed clock models: The *ULNC* and the *nCat2* model. The *ULNC* model is an uncorrelated lognormal clock, which allows each branch rate to be independent of the rates of its ancestors or neighbours. Under this model, the rate of any particular branch is drawn from a lognormal distribution, with the mean and standard deviation of this distribution estimated from the data. We used an exponential prior on the mean rate, *Exp*(10), with a mean of 0.1 and a gamma prior on the s.d. *Gamma*(0.54, 0.38), which has a mean of 0.2. These choices reflect a prior belief that the overall clock rate and variance in the clock rate will both be relatively low but it does not preclude alternative scenarios. The *nCat2* model allows branches to be assigned to different rate categories, in this case two rate categories. The rate category values and assignments are estimated. For the prior on the rate values, we used an exponential distribution, *Exp*(5), with a mean of 0.2. We used a uniform prior on the rate assignments, meaning each branch has a uniform probability of being assigned to either rate category. In both cases, we assume that the overall clock rate was shared across traits. Due to the independence in clock rates, it is possible for these kinds of clock models to account for relatively accelerated bursts of morphological evolution along a given branch.

#### 2.3.4. Skyline analysis

To estimate diversification (birth, death) and sampling rates in different time intervals, we used the FBD skyline model from the BDSky package [56] in BEAST2. This model allows for piecewise constant rate variation through time, meaning that discrete intervals have independent rates. For the skyline models, we chose interval boundaries at 14 600, 12 900 and 11 700 BP to compare the rates between the period before the end of the Last Glacial Maximum (GS−2), the Late Glacial Interstadial (GI−1), the Younger Dryas (GS−1) and the early Holocene, respectively.

We parametrized the model using the birth (λ), death (μ) and sampling rates (ψ), as described above, and calculated the diversification (*d*) and turnover (*t*) rates post hoc. The diversification rate is the rate at which the tree grows and can be defined as


d=λ−μ.

The turnover rate measures the rate at which lineages are replaced and can be define as


$$t=\frac{\mu }{\lambda }.$$

Note that, *d* is within the interval (–∞, ∞) and *t* is within the interval (0, ∞). We calculated the median of the estimated 95% highest posterior density (HPD) intervals for all rates (λ, μ, ψ, *d*, *t*) within each time bin using the coda R package [96].

#### 2.3.5. Specimen ages

As the specimens’ starting ages and/or fixed ages we chose the median age of each specimen, derived from their respective calibrated radiocarbon SPDs. To account for age uncertainty associated with specimens, we applied a uniform prior between the oldest and youngest radiometric dates of the one-sigma age range from the SPD of the calibrated radiocarbon dates collected from each artefact’s respective layer, as described above. An overview of the different scripts is given in the electronic supplementary material, table S1. All ages were divided by 1000 and the results rounded to three decimals.

#### 2.3.6. Testing different combinations of taxa and traits

To study the influence of the number of taxa and traits on the clock rates, we ran several different combinations of taxa and traits. For this, we created four different sets of taxa. One set contains all available artefacts (87 taxa), and the other three datasets have been subsampled in a stratified way, to contain comparable numbers of taxa per chronological interval (4, 8 and 16 per event), resulting in 16, 32 and 60 taxa, in addition to the dataset with 87 taxa. For each of these sets, we experimented with the number of traits and selected the first 2, 3, 6, 9, 10, 20, 44 (corresponding to 50% of the total amount of PC axes rounded up) and all PC axes (87) as traits. As figure 4*a* shows, 99% of the dataset’s total variation is described by the first nine PCs, underlining the importance of experimenting with variable trait numbers. Beyond the convenience that decreasing the number of traits reduces computing time until convergence, importantly, a recent study [97] has also shown that the inclusion of more principal components can lead to an increase in noise and an attendant reduction in phylogenetic signal.

#### 2.3.7. Markov chain Monte Carlo settings

For each script, we ran two independent Markov chain Monte Carlo (MCMC) chains on a high-performance computing system for as many generations as it took to arrive at prior, posterior and likelihood effective sample size (ESS) values all above 200. An ESS of 200 corresponds to a standard error of the mean of 1.77% of the interval width [98]. The ESS value was calculated and convergence assessed using the coda package [96] in R, and Tracer [99]. Depending on the number of taxa, traits, moves and model specifications, MCMC analyses had to run—for each independent run—for a range between 348 500 000 and 22 820 000 000 generations before convergence was reached. Using logcombiner [8], we collated the log-files and the tree-files of each pair of converged, independent chains, with a 20% burn-in. All further analyses were conducted using the combined log- and tree-files.

To quantify and compare the influence of the different taxa–trait combinations on the clock rates, we visualized the median clock rate of the *ULNC* model, as well as the variance in the overall clock rate, on a taxa–trait grid using ggplot2 [100] (electronic supplementary material, figure S1; for the *nCat2* model, see electronic supplementary material, figure S2).

#### 2.3.8. Running the models under the prior

For 16, 32 and 87 taxa, we ran the *ULNC* skyline model with age uncertainty ‘under the prior’, which means we can obtain estimates of the diversification rates under the FBD model, while excluding the influence of the trait and clock models. By excluding the trait information, we are able to distinguish the signal that comes from the fossil sampling times, in the absence of any information from the traits.

#### 2.3.9. Phylogenetic trees

**Figure 6.**
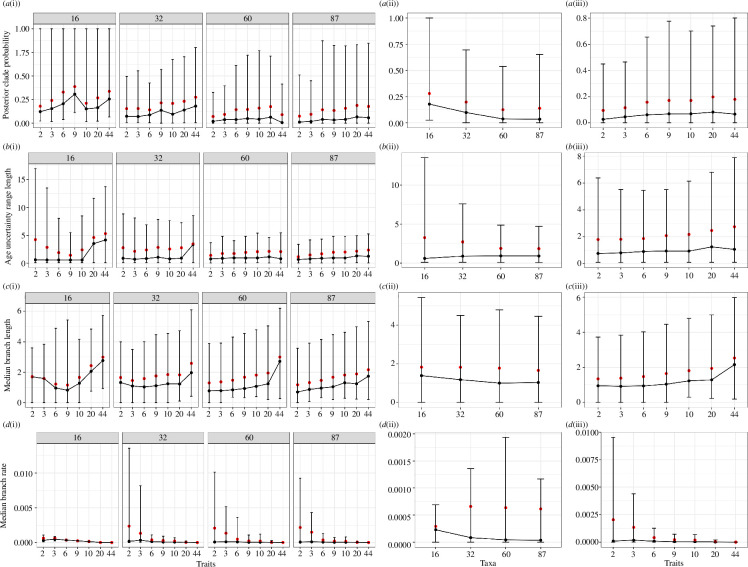
Metrics derived from each MCC tree based on the *ULNC* skyline model with age uncertainty. (*a*) The posterior clade probabilities, (*b*) the lengths of the 95% HPD range of the node heights, (*c*) the median branch lengths, and (*d*) the median branch rates. In sub-plots (i), the data are shown for each trait set (x-axis), grouped by the number of taxa (16, 32, 60, 87). In (ii), the plot shows all values separated by taxa, summarizing the values across traits. In (iii), all values are separated by trait sets, summarizing the values across taxa. The black dots represent the median, and the red dots the mean. The HPD intervals are represented as vertical bars.

Using the package TreeAnnotator [8], we calculated a maximum clade credibility (MCC) tree from each combined output tree file keeping the target heights. Based on the MCC trees, we extracted various metrics such as posterior clade probabilities (PP), median branch lengths and rates, and the lengths of the 95% HPD range of the node heights representing age uncertainty. We visualized these metrics across all different taxa–trait combinations for the skyline model with age uncertainty under the *ULNC* model (figure 6).

## 3. Results

### 3.1. Increasing traits based on principal component axes decreases the evolutionary rate and variance estimates

For complex phylodynamic inference, every parameter and prior choice requires careful consideration, especially in the context of novel applications. We therefore tested how analyses using continuous traits behaved in relation to the different parameters and model choices, and to investigate, whether different models affect our ability to test certain hypotheses. Electronic supplementary material figures S1A and S2A, show that for the *ULNC* and *nCat2* models respectively, the median clock rate (the overall rate at which traits evolve over time) is reduced with the addition of more traits. The highest median rates are recovered—with a few exceptions—from the dataset of 32 taxa and two traits. Electronic supplementary material, figure S1B, shows the clock rate variance (i.e. the degree to which rates vary across lineages) estimated under the *ULNC* relaxed clock model (see electronic supplementary material, figure S2B, for the *nCat2* clock model). Here, the variance is highest for the subset containing 60 taxa and two traits. Meanwhile, the variance is highest for the smallest number of traits used (three for the *ULNC* model). The lowest variance in rates is recovered for the smallest subset of 16 taxa. We conclude that more traits contribute to an overall slower average rate of change. These patterns match what we would expect from figure 4*a*, where each additional trait (PC) adds less and less variation to the data.

Using the *ULNC* skyline model with age uncertainty, figure 6 summarizes different metrics derived from the MCC tree model across the different taxa–trait combinations. Figure 6(*a*(i)–(iii)) shows that the low PP values make it challenging to recover support for any specific topology. Further, the limited increase or even decrease in support with the addition of more taxa is partly attributed to the low PP values, resulting in fewer recovered monophyletic groups across the posterior. The observation of a slight increase with $n_{\text{taxa}}=9$ may be a result of semi-randomly subsampled taxa, but confirmation and further understanding would require simulations. The sub-plots in figure 6(*b*(i)–(iii)) visualize the length of the age uncertainty range. With an increase in taxa, the overall mean age uncertainty decreases, as well as the variance (figure 6(*b*(ii))). The addition of traits has the opposite effect because the clock rates also decrease with the addition of more traits (figure 6(*b*(iii))). The addition of traits seems to be particularly influential for datasets with few taxa (figure 6(*b*(i))). Median branch lengths increase with the addition of traits, yet the overall median branch rates decrease with the addition of taxa. Here too, the addition of traits seems to be particularly influential for datasets with few taxa (figure 6(*c*(i))). The addition of traits reduces both the median branch rates and their variance (figure 6(*d*(iii))). Overall, median branch rates are higher for datasets with more taxa (figure 6(*d*(i)*,*(ii))).

**Figure 7.**
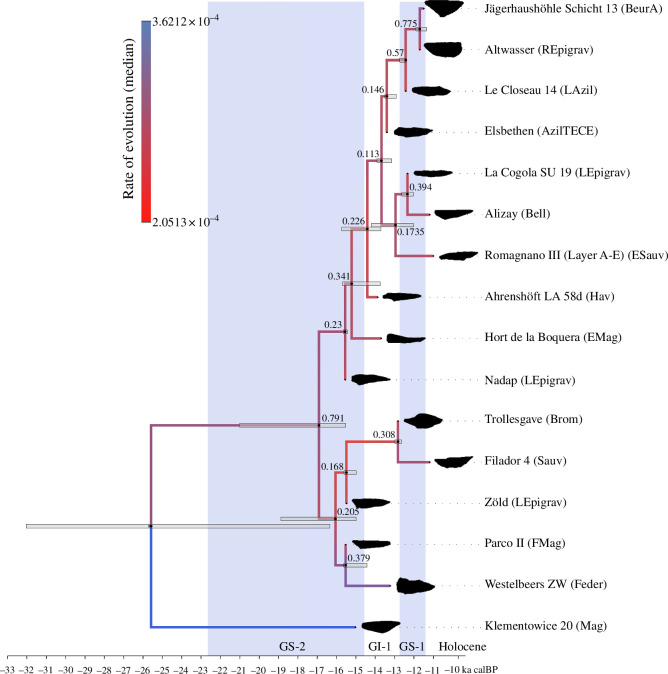
Time-scaled MCC tree of the dataset with 16 taxa and the first nine PC axes as traits using the skyline model with age uncertainty and the *ULNC* clock model. The edges are coloured according to their median clock rate. The horizontal grey bars show the 95% HPD of the ages. PP values are shown for each clade. For readability, the tip labels state the name of the site and the abbreviated archaeological taxonomic unit to which they traditionally are assigned, derived from the original dataset. Sampled ancestors/zero length-edges are not collapsed. The tree was created using FigTree [101] and modified with the Inkscape software package [102].

Figure 7 shows the MCC tree under the *ULNC* skyline model with age uncertainty. For illustrative purposes, the dataset containing 16 taxa and nine traits was chosen. Like all other trees inferred for this study, posterior clade probabilities are low. Nonetheless, the advantage of the Bayesian inference of phylogenies—as conducted here—is that despite topological uncertainty we can use the data to analyse rates of change in a skyline analysis.

### 3.2. Skyline analysis and rates of cultural evolution

**Figure 8.**
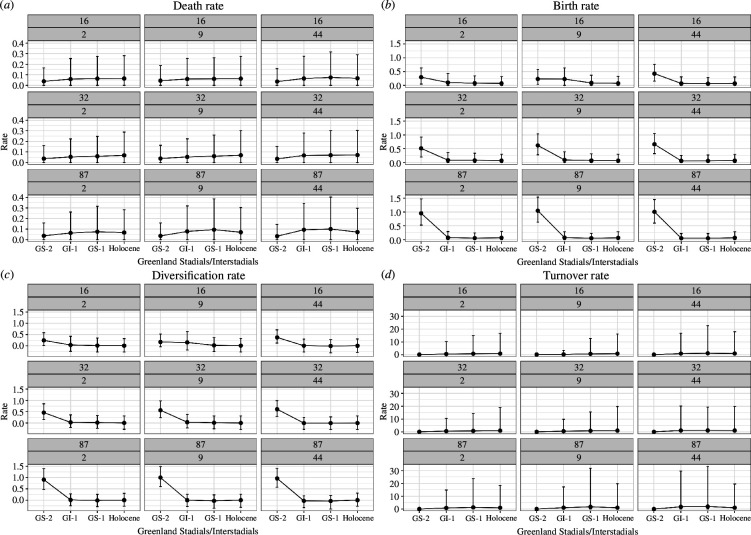
Results of the skyline analysis with age uncertainty using the *ULNC* model for 16, 32 and 87 taxa with 2, 9 and 44 traits. Visualized are the 95% HPD intervals and medians for the (*a*) death, (*b*) birth, (*c*) diversification and (*d*) turnover rates for the four major climatic periods of the Greenland ice-core event stratigraphy [57]. The scale of the y-axes differs between each of the four sub-plots.

**Figure 9.**
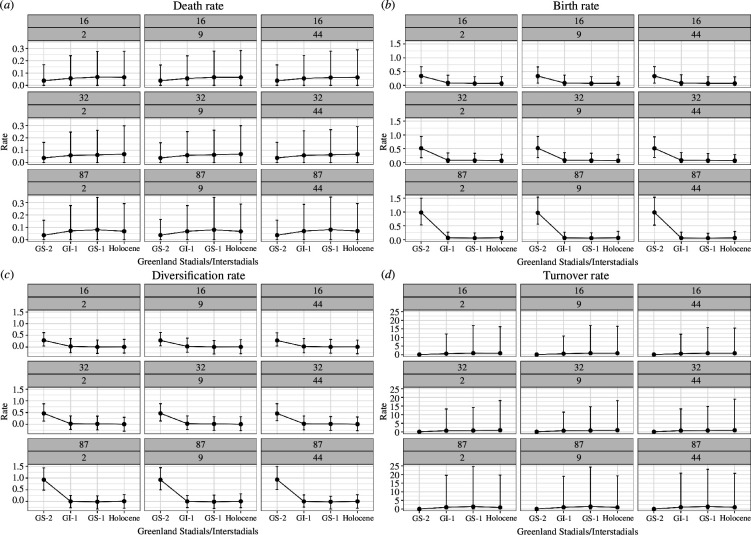
Results from under the prior of the skyline analysis with age uncertainty using the *ULNC* clock model for 16, 32 and 87 taxa with 2, 9 and 44 traits. Visualized are the 95% HPD intervals and medians for the (*a*) death, (*b*) birth, (*c*) diversification and (*d*) turnover rates for the four major climatic periods of the Greenland ice-core event stratigraphy [57]. See figure 8.

Figure 8 shows the death, birth, diversification and turnover rates for the four chosen climate time bins of the period before the end of GS−2, GI−1, GS−1 and the onset of the Holocene for the different combinations of taxa and traits from the skyline analysis with age uncertainty under the *ULNC* model (for the *nCat2* model, see electronic supplementary material, figure S3). It shows that, although the magnitude of rates varies, the overall trend is stable across all trait combinations. Note the early decline in the birth rate for all taxa–trait combinations in GI−1, except for the model using 16 taxa and nine traits, where the birth rate first drops in the time bin GS−1. When comparing the death rates between the different subsets of taxa, the median death rates and their variance are highest in GI−1 for the subset of 87 taxa, where they decrease again in the Holocene. These general tendencies can also be found when running the same models under the prior, which allows us to exclude the trait information, and study how much signal is generated from the age information *per se* (figure 9). This general consistency indicates that the signal for the FBD model parameters is, in fact, coming from the sampling times. Based on visual inspection alone, the results differ the most from the results obtained under prior for the median birth rates of the subset of 16 taxa and nine traits.

The results of the skyline analysis depict a clear signal, more apparent with the addition of taxa, as the higher birth rate for the period before the end of GS−2 could neither be guessed from the dates of our sites (figure 5) nor from the prior, which was set to an exponential distribution with a mean of 0.1. Hence, the results of the skyline analysis are robust to the number of taxa and traits.

## 4. Discussion

The experimental use of continuous traits for the inference of Bayesian phylogenies under a phylodynamic model (the FBD skyline) presented in this paper is novel in archaeology. Until recently, the phylogenetic study of archaeological artefacts has largely been limited to the inference of trees using either parsimony or maximum likelihood approaches, chiefly relying on discrete and often somewhat arbitrary traits. Not only do we infer a phylogenetic tree using continuous data within a Bayesian framework but we also incorporate information about the age of the artefacts by applying a model that incorporates sampling-through-time, the FBD process. In doing so, we were able to derive macroevolutionary metrics such as birth and death rates through time. The Bayesian framework formalizes such a model-based approach and, importantly, provides a measure of uncertainty for the results.

Given the novel nature of this approach, much effort was put into exploring the impact of the data on the different elements of our model, by adding complexity step-by-step. By benchmarking the different models and the impact of changing numbers of taxa and traits, we found, that—for these data, projectile point shapes from the European LUP in the form of PCs—the addition of many shape aspects (PCs) contributed to an overall decrease in average rates of change and their variances. Our results show that the number of taxa used has an impact on model performance as well. We see the reason behind this in what is visualized in figure 4*a*, where each additional trait (PC) added captures increasingly less variation in the data. Similar observations were made recently in the context of a study using principal components as phylogenetic characters of single-cell gene expression data [97]. In our case, the conclusion to select as few traits as possible for the phylogenetic inference, to arrive at maximal evolutionary rates, and to decrease noise in the data, would, in our view, be premature, however. Choosing the model with the most taxa and the fewest traits, resulting in the highest rates, would unduly limit the sampled design space of the artefacts available, as additional traits describe distinct features of the LUP shape repertoire, such as ‘tanged’, ‘shouldered’ or ‘backed’ artefacts.

The low posterior clade probabilities across our inferred phylogenies overall preclude any firm interpretations based on their topologies. Such topological uncertainty supports the argument that existing archaeological taxonomies of the LUP—often predominantly based on artefact shape—are not well-founded, as empirical studies have shown (e.g. [66]). That said, the broad contrast over time in birth and death rates across changing climates may reflect changing mobility strategies and hence population contact under cold and warm regimes, respectively. By the same token, and as already noted by [40], LUP armature outlines may in general be relatively poorly suited for these kinds of analyses due to the overall simplicity of the shapes and the seeming lack of normativity in form imposition at this time. In contrast, the results of a companion study, using the outline shapes of Late Neolithic/Early Bronze Age arrowheads deliberately shaped in the context of prestige display, achieved overall higher posterior clade probabilities [49]. Phylogenetic analyses of lithic armatures may achieve greater resolution by combining matrices consisting of qualitative traits related to their manufacture and outline shape data. Although horizontal transmission and information sharing across cultural lineages could complicate the phylogenetic signal, it is not possible to test this hypothesis using our modelling framework. Future research examining this aspect of cultural evolution could explore the use of network-based inference tools (e.g. Neighbor-Net [103]). However, network analysis in combination with the FBD model is not currently possible, and using networks to visualise historical relations with considerable time-depth also harbours a range of conceptual challenges (cf. [104]).

Despite the topological uncertainties of the trees, we can legitimately deploy the inferred results to analyse the data at the macro-scale—a key advantage of Bayesian phylodynamic models. Birth and death rates were inferred through a skyline analysis for the four distinct climatological time bins representing warming and cooling events within the temporal scope of this study, that is the end of GS−2, GI−1, GS−1 and the onset of the Holocene. Birth rates were highest at the end of the Last Glacial Maximum (GS−2) and declined in the following time bins. High innovation rates under climate-induced pressure have been observed elsewhere [105,106], while the rapid expansion of hunter-gatherer communities at this time may also have led to isolation by distance. Death rates, in contrast, were lowest in GS−2 and increased in GI−1 and GS−1, before decreasing again at the beginning of the Holocene. The consistent association of artefacts whose traditional labels assign them to the so-called Epigravettian—a southeastern phenomenon—and the Azilian and Federmesser cultures—more northwestern phenomena—aligns well with recent palaeogenomic results [73] that would suggest a genetic relation between precisely those units.

## 5. Conclusion

This paper is the first effort in inferring Bayesian phylogenies from continuous lithic armature outline shape data under a phylodynamic model. The analysis conducted using the FBD skyline model is a first step into the realm of greatly expanded analytical possibilities for studying stone tool evolution in a macro-archaeological way. The reproducible and modular workflow provided here can now be used and expanded in a myriad of ways. For example, future studies could be dedicated to the evaluation of the most likely models of shape evolution and compare models other than Brownian motion [41,107], such as the Ornstein–Uhlenbeck process [108–110] or an Early Burst model [111,112]. Furthermore, it could be tested whether the robustness of the phylogenies changes with the inclusion of additional data, such as biogeographic information, or in combination with other phylogenetic sources, such as language- or ancient DNA-based phylogenies. Combining qualitative technological traits and shape data would also doubtlessly improve phylogenetic inference, although such data remain difficult to obtain in adequately large sample sizes and lingering issues of intra- and inter-observer variability remain [113]. The choice of artefacts could be evaluated, too, and more evidently standardized artefacts than the ones of the LUP, such as the lithic projectile points of the Neolithic, or even pottery profiles could be taken into service. Once robust artefact phylogenies are in place, the extensive analytical repertoire that phylogenetic comparative methods offer, such as testing the association between artefact shape and their size, and/or with favoured prey species as inferred from associated faunal data, could also be taken into use. Finally, the reconstruction of explicit artefact phylodynamics as explored here offers exciting opportunities for analysing genetic and artefactual data in parallel. Both approaches are underwritten by explicit generative models of change—genetic and cultural evolution respectively—and share common methodological features. It might thereby become possible to conduct explicit gene-culture co-evolutionary analyses in these very remote periods of the human past.

## Data Availability

All code and data associated with this paper are available in the accompanying research compendium on Zenodo under [114]. Supplementary material is available online [115].

## References

[1] López-Antoñanzas R, Mitchell J, Simões TR, Condamine FL, Aguilée R, Peláez-Campomanes P, Renaud S, Rolland J, Donoghue PCJ. 2022 Integrative phylogenetics: tools for palaeontologists to explore the tree of life. Biology **11**, 1185. (10.3390/biology11081185) 36009812 PMC9405010

[2] Sumrall CD, Brochu CA. 2008 Viewing paleobiology through the lens of phylogeny. Paleontol. Soc. Pap. **14**, 165–183. (10.1017/S1089332600001674)

[3] Hennig W. 1965 Phylogenetic systematics. Annu. Rev. Entomol. **10**, 97–116. (10.1146/annurev.en.10.010165.000525)

[4] Hagen JB. 2001 The introduction of computers into systematic research in the United States during the 1960s. Stud. Hist. Philos. Sci. C **32**, 291–314. (10.1016/S1369-8486(01)00005-X)

[5] Hagen J. 2003 The statistical frame of mind in systematic biology from quantitative zoology to biometry. J. Hist. Biol. **36**, 353–384. (10.1023/a:1024479322226) 12945573

[6] Drummond AJ, Ho SYW, Phillips MJ, Rambaut A. 2006 Relaxed phylogenetics and dating with confidence. PLoS Biol. **4**, e88. (10.1371/journal.pbio.0040088) 16683862 PMC1395354

[7] Wright AM. 2019 A systematist’s guide to estimating Bayesian phylogenies from morphological data. Insect Syst. Divers. **3**, 2. (10.1093/isd/ixz006) 31355348 PMC6643758

[8] Bouckaert R, Heled J, Kühnert D, Vaughan T, Wu CH, Xie D, Suchard MA, Rambaut A, Drummond AJ. 2014 BEAST 2: a software platform for Bayesian evolutionary analysis. PLoS Comput. Biol. **10**, e1003537. (10.1371/journal.pcbi.1003537) 24722319 PMC3985171

[9] Höhna S, Landis MJ, Heath TA, Boussau B, Lartillot N, Moore BR, Huelsenbeck JP, Ronquist F. 2016 RevBayes: Bayesian phylogenetic inference using graphical models and an interactive model-specification language. Syst. Biol. **65**, 726–736. (10.1093/sysbio/syw021) 27235697 PMC4911942

[10] Atkinson QD, Gray RD. 2005 Curious parallels and curious connections – phylogenetic thinking in biology and historical linguistics. Syst. Biol. **54**, 513–526. (10.1080/10635150590950317) 16051587

[11] Searls DB. 2003 Linguistics: trees of life and of language. Nature **426**, 391–392. (10.1038/426391a) 14647362

[12] Rexová K, Frynta D, Zrzavý J. 2003 Cladistic analysis of languages: Indo-European classification based on lexicostatistical data. Cladistics Int. J. Willi Hennig Soc. **19**, 120–127. (10.1111/j.1096-0031.2003.tb00299.x)

[13] Forster P, Renfrew C. 2006 Phylogenetic methods and the prehistory of languages. Cambridge, UK: McDonald Institute Monographs.

[14] Dunn M, Terrill A, Reesink G, Foley RA, Levinson SC. 2005 Structural phylogenetics and the reconstruction of ancient language history. Science **309**, 2072–2075. (10.1126/science.1114615) 16179483

[15] Hajič Jr J, Ballen GA, Mühlová KH, Vlhová-Wörner H. 2023 Towards building a phylogeny of Gregorian chant melodies. In Proc. of the 24th Int. Society for Music Information Retrieval Conf., Milan, Italy, pp. 571–578. ISMIR. (10.5281/zenodo.10340442)

[16] Marwick B. 2006 What can archaeology do with Boyd and Richerson’s cultural evolutionary program? Rev. Archaeol. **26**, 30–40. https://openresearch-repository.anu.edu.au/items/b715b3b1-2c71-4278-8c37-977d1c5a1a11

[17] Riede F, Araujo A, Marwick B. 2022 Robert C. Dunnell’s Systematics in prehistory at 50. Evol. Hum. Sci. **4**, e16. (10.1017/ehs.2022.18) 35663508 PMC7612791

[18] Rozoy JG. 1978 Les derniers chasseurs: l’Epipaléolithique en France et en Belgique, essai de synthèse. Reims, France: Édition Société Archéologique Champenoise.

[19] Chapa Brunet MT. 1984 Aspectos metodológicos de la tipología arqueológica: un ejemplo referido a las fibulas de la tene. In Primeras jornadas de metodología de investigación prehistórica, pp. 253–268. Madrid, Spain: Instituto de Conservación y Restauración de Bienes Culturales.

[20] Buchanan B, Collard M. 2008 Testing models of early Paleoindian colonization and adaptation using cladistics. In Cultural transmission and archaeology: issues and case studies (ed. MJ O’Brien), pp. 59–76. Washington, DC: Society for American Archaeology Press.

[21] Darwent J, O’Brien MJ. 2006 Using cladistics to construct lineages of projectile points from northeastern Missouri. In Mapping our ancestors (eds CP Lipo, MJ O’Brien, M Collard, SJ Shennan). New Brunswick, NJ: AldineTransaction. (10.4324/9780203786376-12). See https://www.taylorfrancis.com/books/9781351507073.

[22] O’Brien MJ, Buchanan B, Collard M, Boulanger MT. Cultural cladistics and the early prehistory of North America. In Evolutionary biology: mechanisms and trends (ed. P Pontarotti), pp. 23–42. Berlin, Germany: Springer.

[23] O’Brien MJ, Darwent J, Lyman RL. 2001 Cladistics is useful for reconstructing archaeological phylogenies: Palaeoindian points from the southeastern United States. J. Archaeol. Sci. **28**, 1115–1136. (10.1006/jasc.2001.0681)

[24] O’Brien MJ, Lyman RL. 2003 Cladistics and archaeology. Salt Lake City, UT: University of Utah Press.

[25] Tripp A. 2016 A cladistics analysis exploring regional patterning of the anthropomorphic figurines from the Gravettian. In Cultural phylogenetics (ed. L Mendoza Straffon), pp. 179–202. Cham, Switzerland: Springer International Publishing. (10.1007/978-3-319-25928-4_8)

[26] Cardillo M, Charlin J. 2018 Phylogenetic analysis of stemmed points from Patagonia: shape change and morphospace evolution. J. Lithic Stud. **5**, 1–31. (10.2218/jls.2797)

[27] Matthews LJ, Tehrani JJ, Jordan FM, Collard M, Nunn CL. 2011 Testing for divergent transmission histories among cultural characters: a study using Bayesian phylogenetic methods and Iranian tribal textile data. PLoS One **6**, e14810. (10.1371/journal.pone.0014810) 21559083 PMC3084691

[28] Buckley CD. 2012 Investigating cultural evolution using phylogenetic analysis: the origins and descent of the southeast Asian tradition of warp ikat weaving. PLoS One **7**, e52064. (10.1371/journal.pone.0052064) 23272211 PMC3525544

[29] Buckley CD, Boudot E. 2017 The evolution of an ancient technology. R. Soc. Open Sci. **4**, 170208. (10.1098/rsos.170208) 28573032 PMC5451833

[30] Gjesfjeld E, Jordan P. 2019 Contributions of Bayesian phylogenetics to exploring patterns of macroevolution in archaeological data. In Handbook of evolutionary research in archaeology (ed. AM Prentiss), pp. 161–182. Cham, Switzerland: Springer International Publishing. (10.1007/978-3-030-11117-5_9)

[31] Prentiss AM, Walsh MJ, Gjesfjeld E, Denis M, Foor TA. 2022 Cultural macroevolution in the middle to late Holocene Arctic of east Siberia and North America. J. Anthropol. Archaeol. **65**, 101388. (10.1016/j.jaa.2021.101388)

[32] O’Brien MJ, Lyman RL, Mesoudi A, VanPool TL. 2010 Cultural traits as units of analysis. Phil. Trans. R. Soc. B **365**, 3797–3806. (10.1098/rstb.2010.0012) 21041205 PMC2981907

[33] Riede F. 2011 Steps towards operationalising an evolutionary archaeological definition of culture. In Investigating archaeological cultures: material culture, variability, and transmission (eds B Roberts, M Vander Linden), pp. 245–270. New York, NY: Springer. (10.1007/978-1-4419-6970-5_13)

[34] Sober E. 1980 Evolution, population thinking, and essentialism. Philos. Sci. **47**, 350–383. (10.1086/288942)

[35] O’Brien MJ, Lyman RL. 2002 The epistemological nature of archaeological units. Anthropol. Theory **2**, 37–56. (10.1177/1463499602002001287)

[36] Bailey K. 1994 Typologies and taxonomies. Thousand Oaks, CA: SAGE Publications, Inc. (10.4135/9781412986397)

[37] Cardillo M. 2010 Some applications of geometric morphometrics to archaeology. In Morphometrics for nonmorphometricians (ed. AMT Elewa), pp. 325–341. Berlin, Germany: Springer. (10.1007/978-3-540-95853-6_15)

[38] Okumura M, Araujo AGM. 2014 Long-term cultural stability in hunter-gatherers: a case study using traditional and geometric morphometric analysis of lithic stemmed bifacial points from Southern Brazil. J. Archaeol. Sci. **45**, 59–71. (10.1016/j.jas.2014.02.009)

[39] Lycett SJ, von Cramon-Taubadel N, Foley RA. 2006 A crossbeam co-ordinate caliper for the morphometric analysis of lithic nuclei: a description, test and empirical examples of application. J. Archaeol. Sci. **33**, 847–861. (10.1016/j.jas.2005.10.014)

[40] Matzig DN, Hussain ST, Riede F. 2021 Design space constraints and the cultural taxonomy of European Final Palaeolithic large tanged points: a comparison of typological, landmark-based and whole-outline geometric morphometric approaches. J. Paleo. Arch. **4**, 27. (10.1007/s41982-021-00097-2)

[41] Felsenstein J. 1973 Maximum-likelihood estimation of evolutionary trees from continuous characters. Am. J. Hum. Genet. **25**, 471–492. 4741844 PMC1762641

[42] Felsenstein J. 1988 Phylogenies and quantitative characters. Annu. Rev. Ecol. Syst. **19**, 445–471. (10.1146/annurev.es.19.110188.002305)

[43] Goloboff PA, Mattoni CI, Quinteros AS. 2006 Continuous characters analyzed as such. Cladistics Int. J. Willi Hennig Soc. **22**, 589–601. (10.1111/j.1096-0031.2006.00122.x) 34892898

[44] Rae TC. 1998 The logical basis for the use of continuous characters in phylogenetic systematics. Cladistics Int. J. Willi Hennig Soc. **14**, 221–228. (10.1111/j.1096-0031.1998.tb00335.x) 34905828

[45] Smith UE, Hendricks JR. 2013 Geometric morphometric character suites as phylogenetic data: extracting phylogenetic signal from gastropod shells. Syst. Biol. **62**, 366–385. (10.1093/sysbio/syt002) 23325808

[46] Parins-Fukuchi C. 2018 Bayesian placement of fossils on phylogenies using quantitative morphometric data. Evolution **72**, 1801–1814. (10.1111/evo.13516) 29998561

[47] Parins-Fukuchi C. 2018 Use of continuous traits can improve morphological phylogenetics. Syst. Biol. **67**, 328–339. (10.1093/sysbio/syx072) 28945906

[48] Álvarez-Carretero S, Goswami A, Yang Z, Dos Reis M. 2019 Bayesian estimation of species divergence times using correlated quantitative characters. Syst. Biol. **68**, 967–986. (10.1093/sysbio/syz015) 30816937

[49] Marwick B, Matzig DN, Riede F. 2023 Bayesian inference of material culture phylogenies using continuous traits: a birth–death model for Late Neolithic/Early Bronze Age arrowheads from Northwestern Europe. SocArXiv. (10.31235/osf.io/j2kva)

[50] Stadler T. 2010 Sampling-through-time in birth–death trees. J. Theor. Biol. **267**, 396–404. (10.1016/j.jtbi.2010.09.010) 20851708

[51] Heath TA, Huelsenbeck JP, Stadler T. 2014 The fossilized birth–death process for coherent calibration of divergence-time estimates. Proc. Natl Acad. Sci. USA **111**, E2957–E2966. (10.1073/pnas.1319091111) 25009181 PMC4115571

[52] Zhang H, Ji T, Pagel M, Mace R. 2020 Dated phylogeny suggests early Neolithic origin of Sino-Tibetan languages. Sci. Rep. **10**, 20792. (10.1038/s41598-020-77404-4) 33247154 PMC7695722

[53] Grenfell BT, Pybus OG, Gog JR, Wood JLN, Daly JM, Mumford JA, Holmes EC. 2004 Unifying the epidemiological and evolutionary dynamics of pathogens. Science **303**, 327–332. (10.1126/science.1090727) 14726583

[54] Stadler T, Kühnert D, Bonhoeffer S, Drummond AJ. 2013 Birth–death skyline plot reveals temporal changes of epidemic spread in HIV and hepatitis C virus (HCV). Proc. Natl Acad. Sci. USA **110**, 228–233. (10.1073/pnas.1207965110) 23248286 PMC3538216

[55] Nadeau SA, Vaughan TG, Scire J, Huisman JS, Stadler T. 2021 The origin and early spread of SARS-CoV-2 in Europe. Proc. Natl Acad. Sci. USA **118**, e2012008118. (10.1073/pnas.2012008118) 33571105 PMC7936359

[56] Gavryushkina A, Welch D, Stadler T, Drummond AJ. 2014 Bayesian inference of sampled ancestor trees for epidemiology and fossil calibration. PLoS Comput. Biol. **10**, e1003919. (10.1371/journal.pcbi.1003919) 25474353 PMC4263412

[57] Rasmussen SO *et al*. 2014 A stratigraphic framework for abrupt climatic changes during the Last Glacial period based on three synchronized Greenland ice-core records: refining and extending the INTIMATE event stratigraphy. Quat. Sci. Rev. **106**, 14–28. (10.1016/j.quascirev.2014.09.007)

[58] Straus LG, Eriksen BV, Erlandson JM, Yesner DR. 1996 Humans at the end of the Ice Age: the archaeology of the Pleistocene–Holocene transition. New York, NY: Plenum Press. (10.1007/978-1-4613-1145-4)

[59] Erin MI. 2012 On Younger Dryas climate change as a causal determinate of prehistoric hunter-gatherer culture change. In Hunter-gatherer behavior: human response during the Younger Dryas (ed. MI Erin), pp. 11–23. Walnut Creek, CA: Left Coast Press.

[60] Terberger T, Eriksen BV. 2004 Hunters in a changing world: environment and archaeology of the Pleistocene–Holocene transition (ca. 11000-9000 B.C.) in Northern Central Europe. In Workshop of the U.I.S.P.P.-commission XXXII at Greifswald in September 2002. Rahden, Westphalia: Verlag Marie Leidorf.

[61] Straus LG. 1996 The archaeology of the Pleistocene–Holocene transition in Southwest Europe. In Humans at the end of the Ice Age: the archaeology of the Pleistocene–Holocene transition (eds LG Straus, BV Eriksen, JM Erlandson, DR Yesner), pp. 83–99. New York, NY: Plenum Press.

[62] Perreault C. 2019 The quality of the archaeological record. Chicago, IL: University of Chicago Press.

[63] Ivanovaitė L, Serwatka K, Hoggard CS, Sauer F, Riede F. 2020 All these fantastic cultures? Research history and regionalization in the Late Palaeolithic tanged point cultures of Eastern Europe. Eur. J. Archaeol. **23**, 1–24. (10.1017/eaa.2019.59)

[64] Sauer F, Riede F. 2019 A critical reassessment of cultural taxonomies in the Central European Late Palaeolithic. J. Archaeol. Method. Theory **26**, 155–184. (10.1007/s10816-018-9368-0)

[65] Kobusiewicz M. 2009 Whether the Bromme culture existed? Folia. Praehist. Posn. **XV**, 75–91. http://hdl.handle.net/10593/9533

[66] Riede F *et al*. 2024 A quantitative analysis of Final Palaeolithic/earliest Mesolithic cultural taxonomy and evolution in Europe. PLoS One **19**, e0299512. (10.1371/journal.pone.0299512) 38466685 PMC10927100

[67] Matzig DN, Schmid C, Riede F. 2023 Mapping the field of cultural evolutionary theory and methods in archaeology using bibliometric methods. Humanit. Soc. Sci. Commun. **10**, 1–17. (10.1057/s41599-023-01767-y)

[68] Reynolds N, Riede F. 2019 House of cards: cultural taxonomy and the study of the European Upper Palaeolithic. Antiquity **93**, 1350–1358. (10.15184/aqy.2019.49)

[69] Hussain ST *et al*. 2023 A pan-European dataset revealing variability in lithic technology, toolkits, and artefact shapes ~15-11 kya. Sci. Data **10**, 593. (10.1038/s41597-023-02500-9) 37679390 PMC10484899

[70] Jordan P. 2015 Technology as human social tradition: cultural transmission among hunter-gatherers. Berkeley, CA: University of California Press.

[71] Manem S. 2020 Modeling the evolution of ceramic traditions through a phylogenetic analysis of the chaînes opératoires: the European Bronze Age as a case study. J. Archaeol. Method Theory **27**, 992–1039. (10.1007/s10816-019-09434-w)

[72] Guillerme T *et al*. 2020 Disparities in the analysis of morphological disparity. Biol. Lett. **16**, 20200199. (10.1098/rsbl.2020.0199) 32603646 PMC7423048

[73] Posth C *et al*. 2023 Palaeogenomics of Upper Palaeolithic to Neolithic European hunter-gatherers. Nature **615**, 117–126. (10.1038/s41586-023-05726-0) 36859578 PMC9977688

[74] Eriksen BV. 2000 Patterns of ethnogeographic variability in Late Pleistocene Western Europe. In Regional approaches to adaptation in Late Pleistocene Western Europe (eds GL Peterkin, HA Price), pp. 147–168. Oxford, UK: Oxbow.

[75] Wiessner P. 1983 Style and social information in Kalahari San projectile points. Am. Antiq. **48**, 253–276. (10.2307/280450)

[76] Wiessner P. 1997 Seeking guidelines through an evolutionary approach: style revisited among the ! Kung San (Ju/’hoansi) of the 1990s. Archeol. Pap. Am. Anthropol. Assoc. **7**, 157–176. (10.1525/ap3a.1997.7.1.157)

[77] Sinopoli CM. 1991 Style in arrows: a study of an ethnographic collection from the Western United States. In Foragers in context: long-term, regional and historical perspectives in hunter-gatherer studies (eds PT Miracle, LE Fisher, J Brown), pp. 63–87. Ann Arbor, MI: University of Michigan Press.

[78] Fischer A. 1990 A Late Palaeolithic ‘school’ of flint-knapping at Trollesgave, Denmark. Results from refitting. Acta. archaeol. **60**, 33–49.

[79] Roberts A, Barton N. 2021 An example of novice flintknapping in the British Late Upper Palaeolithic? In The beef behind all possible pasts: the tandem Festschrift in honour of Elaine Turner and Martin Street (eds S Gaudzinski-Windheuser, O Jöris), pp. 535–546. Heidelberg, Germany: Propylaeum. (10.11588/propylaeum.950)

[80] Bodu P, Karlin C, Ploux S. 1990 Who’s who? the Magdalenian flintknappers of Pincevent, France. In The Big Puzzle: Int. Symp. on Refitting Stone Artefacts, Monrepos, 1987 (eds E Cziesla, S Eickhoff, N Arts, D Winter), pp. 143–163. Bonn, Germany: Holos.

[81] Grimm L. 2000 Apprentice flintknapping: relating material culture and social practice in the Upper Palaeolithic. In Children and material culture (ed. JS Derevenski), pp. 53–71. London, UK: Routledge.

[82] Tostevin GB. 2011 Special issue: Reduction sequence, chaîne opératoire, and other methods: the epistemologies of different approaches to lithic analysis – Introduction. PalaeoAnthropology **2011**, 293–296.

[83] Riede F, Hoggard C, Shennan SJ. 2019 Reconciling material cultures in archaeology with genetic data requires robust cultural evolutionary taxonomies. Palgrave Commun. **5**, 55. (10.1057/s41599-019-0260-7)

[84] Lycett SJ. 2015 Cultural evolutionary approaches to artifact variation over time and space: basis, progress, and prospects. J. Archaeol. Sci. **56**, 21–31. (10.1016/j.jas.2015.01.004)

[85] R Core Team. 2023 *R: A language and environment for statistical computing*. Vienna, Austria: R Foundation for Statistical Computing.

[86] Bonhomme V, Picq S, Gaucherel C, Claude J. 2014 Momocs: outline analysis using R. J. Stat. Softw. **56**, 1–24. (10.18637/jss.v056.i13)

[87] Vermeersch PM. 2023 Radiocarbon Palaeolithic Europe Database, Version 30. See https://www.researchgate.net/project/Radiocarbon-Palaeolithic-Europe-Database. 10.1016/j.dib.2020.105793 PMC7300123 32577447

[88] Crema ER, Bevan A. 2021 Inference from large sets of radiocarbon dates: software and methods. Radiocarbon **63**, 23–39. (10.1017/RDC.2020.95)

[89] Reimer PJ *et al*. 2020 The IntCal20 Northern Hemisphere radiocarbon age calibration curve (0–55 cal kBP). Radiocarbon **62**, 725–757. (10.1017/RDC.2020.41)

[90] Thorne JL, Kishino H, Painter IS. 1998 Estimating the rate of evolution of the rate of molecular evolution. Mol. Biol. Evol. **15**, 1647–1657. (10.1093/oxfordjournals.molbev.a025892) 9866200

[91] Warnock RCM, Wright AM. 2020 Understanding the tripartite approach to Bayesian divergence time estimation. Cambridge, UK: Cambridge University Press. (10.1017/9781108954365)

[92] Smiley TM. 2018 Detecting diversification rates in relation to preservation and tectonic history from simulated fossil records. Paleobiology **44**, 1–24. (10.1017/pab.2017.28)

[93] Černý D, Madzia D, Slater GJ. 2021 Empirical and methodological challenges to the model-based inference of diversification rates in extinct clades. Syst. Biol. **71**, 153–171. (10.1093/sysbio/syab045) 34110409

[94] Allen BJ, Volkova Oliveira MV, Stadler T, Vaughan TG, Warnock RCM. 2024 Mechanistic phylodynamic models do not provide conclusive evidence that non-avian dinosaurs were in decline before their final extinction. Camb. Prisms Extinction **2**, e6. (10.1017/ext.2024.5) PMC11895757 40078801

[95] Zhang R, Drummond AJ, Mendes FK. 2024 Fast Bayesian inference of phylogenies from multiple continuous characters. Syst. Biol. **73**, syad067. (10.1093/sysbio/syad067) PMC11129596 38085256

[96] Plummer M, Best N, Cowles K, Vines K. 2006 CODA: convergence diagnosis and output analysis for MCMC. R. News **6**, 7–11. https://journal.r-project.org/articles/RN-2006-002/RN-2006-002.pdf

[97] Mah JL, Dunn CW. 2024 Cell type evolution reconstruction across species through cell phylogenies of single-cell RNA sequencing data. Nat. Ecol. Evol. **8**, 325–338. (10.1038/s41559-023-02281-9) 38182680

[98] Fabreti LG, Höhna S. 2022 Convergence assessment for Bayesian phylogenetic analysis using MCMC simulation. Methods Ecol. Evol. **13**, 77–90. (10.1111/2041-210X.13727)

[99] Rambaut A, Drummond AJ, Xie D, Baele G, Suchard MA. 2018 Posterior summarization in Bayesian phylogenetics using Tracer 1.7. Syst. Biol. **67**, 901–904. (10.1093/sysbio/syy032) 29718447 PMC6101584

[100] Wickham H. 2016 ggplot2: elegant graphics for data analysis. Cham, Switzerland: Springer-Verlag New York.

[101] Rambaut A. 2018 FigTree v1.4.4. See http://tree.bio.ed.ac.uk/.

[102] Inkscape Project. 2018 Inkscape. See https://inkscape.org.

[103] Bryant D. Neighbor-net: an agglomerative method for the construction of phylogenetic networks. Mol. Biol. Evol. **21**, 255–265. (10.1093/molbev/msh018) 14660700

[104] Fernández-López de Pablo J, Romano V, Derex M, Gjesfjeld E, Gravel-Miguel C, Hamilton MJ, Migliano AB, Riede F, Lozano S. 2022 Understanding hunter-gatherer cultural evolution needs network thinking. Trends Ecol. Evol. **37**, 632–636. (10.1016/j.tree.2022.04.007) 35659425

[105] Fitzhugh B. 2001 Risk and invention in human technological evolution. J. Anthropol. Archaeol. **20**, 125–167. (10.1006/jaar.2001.0380)

[106] Ordonez A, Riede F. 2022 Changes in limiting factors for forager population dynamics in Europe across the last Glacial-Interglacial transition. Nat. Commun. **13**, 5140. (10.1038/s41467-022-32750-x) 36068206 PMC9448755

[107] Mitov V, Bartoszek K, Asimomitis G, Stadler T. 2020 Fast likelihood calculation for multivariate Gaussian phylogenetic models with shifts. Theor. Popul. Biol. **131**, 66–78. (10.1016/j.tpb.2019.11.005) 31805292

[108] Hansen TF, Martins EP. 1996 Translating between microevolutionary process and macroevolutionary patterns: the correlation structure of interspecific data. Evolution **50**, 1404–1417. (10.1111/j.1558-5646.1996.tb03914.x) 28565714

[109] Hansen TF. 1997 Stabilizing selection and the comparative analysis of adaptation. Evolution **51**, 1341–1351. (10.1111/j.1558-5646.1997.tb01457.x) 28568616

[110] Butler MA, King AA. 2004 Phylogenetic comparative analysis: a modeling approach for adaptive evolution. Am. Nat. **164**, 683–695. (10.1086/426002) 29641928

[111] Harmon LJ, Weir JT, Brock CD, Glor RE, Challenger W. 2008 GEIGER: investigating evolutionary radiations. Bioinformatics **24**, 129–131. (10.1093/bioinformatics/btm538) 18006550

[112] Harmon LJ *et al*. 2010 Early bursts of body size and shape evolution are rare in comparative data. Evolution **64**, 2385–2396. (10.1111/j.1558-5646.2010.01025.x) 20455932

[113] Darmark K, Apel J. 2008 The dogma of immaculate perception. an experimental study of bifacial arrowheads and a contribution to the discussion on the relationship between personal experience and formalised analysis in experimental archaeology (eds M M Sørensen, P Desrosiers). In Technology in archaeology. Proc. of the SILA workshop: the study of technology as a method for gaining insight into social and cultural aspects of prehistory: The National Museum of Denmark, Copenhagen, pp. 173–186. Copenhagen, Denmark: Nationalmuseet.

[114] Matzig DN, Marwick B, Riede F, Warnock RCM. 2024 Research compendium for: A macroevolutionary analysis of European Late Upper Palaeolithic stone tool shape using a Bayesian phylodynamic framework. Zenodo. (10.5281/zenodo.10693325) PMC11321859 39144489

[115] Matzig DN, Marwick B, Riede F, Warnock RCM. 2024 Data from: A macroevolutionary analysis of European Late Upper Palaeolithic stone tool shape using a Bayesian phylodynamic framework. Figshare. (10.6084/m9.figshare.c.7389784) PMC11321859 39144489

